# Colorimetric RT-LAMP SARS-CoV-2 diagnostic sensitivity relies on color interpretation and viral load

**DOI:** 10.1038/s41598-021-88506-y

**Published:** 2021-04-27

**Authors:** Mateus Nóbrega Aoki, Bruna de Oliveira Coelho, Luiz Gustavo Bentim Góes, Paola Minoprio, Edison Luiz Durigon, Luis Gustavo Morello, Fabricio Klerynton Marchini, Irina Natassja Riediger, Maria do Carmo Debur, Helder I. Nakaya, Lucas Blanes

**Affiliations:** 1 Laboratory for Applied Science and Technology in Health, Carlos Chagas Institute, Oswaldo Cruz Foundation (Fiocruz), Curitiba, 81310-020 Brazil; 2 Scientific Platform Pasteur - University of São Paulo, São Paulo, 05508-020 Brazil; 3 Departamento de Microbiologia – ICB‐II, Universidade de São Paulo, São Paulo, 05508-000 Brazil; 4 Department of Clinical and Toxicological Analyses, School of Pharmaceutical Sciences, University of São Paulo, São Paulo, 05508-000 Brazil; 5 Paraná´s Central Laboratory (LACEN-PR), Curitiba, 80045-150 Brazil

**Keywords:** Molecular biology, Molecular medicine

## Abstract

The use of RT-LAMP (reverse transcriptase—loop mediated isothermal amplification) has been considered as a promising point-of-care method to diagnose COVID-19. In this manuscript we show that the RT-LAMP reaction has a sensitivity of only 200 RNA virus copies, with a color change from pink to yellow occurring in 100% of the 62 clinical samples tested positive by RT-qPCR. We also demonstrated that this reaction is 100% specific for SARS-CoV-2 after testing 57 clinical samples infected with dozens of different respiratory viruses and 74 individuals without any viral infection. Although the majority of manuscripts recently published using this technique describe only the presence of two-color states (pink = negative and yellow = positive), we verified by naked-eye and absorbance measurements that there is an evident third color cluster (orange), in general related to positive samples with low viral loads, but which cannot be defined as positive or negative by the naked eye. Orange colors should be repeated or tested by RT-qPCR to avoid a false diagnostic. RT-LAMP is therefore very reliable for samples with a RT-qPCR Ct < 30 being as sensitive and specific as a RT-qPCR test. All reactions were performed in 30 min at 65 °C. The use of reaction time longer than 30 min is also not recommended since nonspecific amplifications may cause false positives.

## Introduction

The SARS-CoV-2 virus is the causative agent of the coronavirus disease 2019 (COVID-19) pandemic^1^. According to the World Health Organization (WHO), more than 60 million people have been infected and 1, 416, 101 have died as of November, 2020^2^. At the moment, with few available vaccines or specific drugs for COVID-19 treatment, the best way to control the disease is through strong public health surveillance and rapid diagnostic testing^3,4^. Real-time reverse transcription-PCR (RT-qPCR) assays are being used as the gold standard to diagnose this disease^5^. Despite its high sensitivity and specificity, RT-qPCR requires highly trained personnel, special facilities, and high-cost instrumentation. These factors limit its application despite the high testing demand, especially in underdeveloped countries^6^. Reverse transcription loop-mediated isothermal amplification (RT-LAMP) is an isothermal nucleic acid amplification technique that is being widely tested for detecting infectious diseases, including COVID-19. The method has great potential as a point-of-care tool because it is a rapid, sensitive, and specific technique^7^. LAMP requires a set of four to six primers (which ensures high specificity) and takes less than one hour for amplifying the genetic material of the pathogen^8^. The results of the amplification can be confirmed using different methods, such as changes in turbidity caused by magnesium pyrophosphate precipitate^9^, changes in fluorescence using intercalating dyes, DNA probes with gold nanoparticles^10,11^, pH indicators, or gel electrophoresis followed by UV detection^12^. Among these, the most frequently used method to detect COVID-19 involves using pH-based colorimetric kits with results visible to the naked eye^12–14^. RT-LAMP tests have been previously described as applicable for detecting SARS-CoV-2^12–16^. However, the performance of these tests has not been ideal. In one of these methods^15^, for example, a sample of water was used as a negative control instead of human RNA, which could have led to a false negative result. Other tests failed to evaluate a considerable number of clinical samples^13^ or the specificity of the primers when testing human samples infected with a wide range of other viruses^12,14,16^. In the present study, we benchmarked the previous sets of primers for RT-LAMP (using human RNA as negative control) and we found that the method is as specific as RT-qPCR. We also found that the method is specific after testing it with dozens of other respiratory-related viruses. Further, we discuss the presence of a third color cluster never described before that could lead to a wrong diagnosis.

## Results

### LAMP primers screening

**Figure 1:**
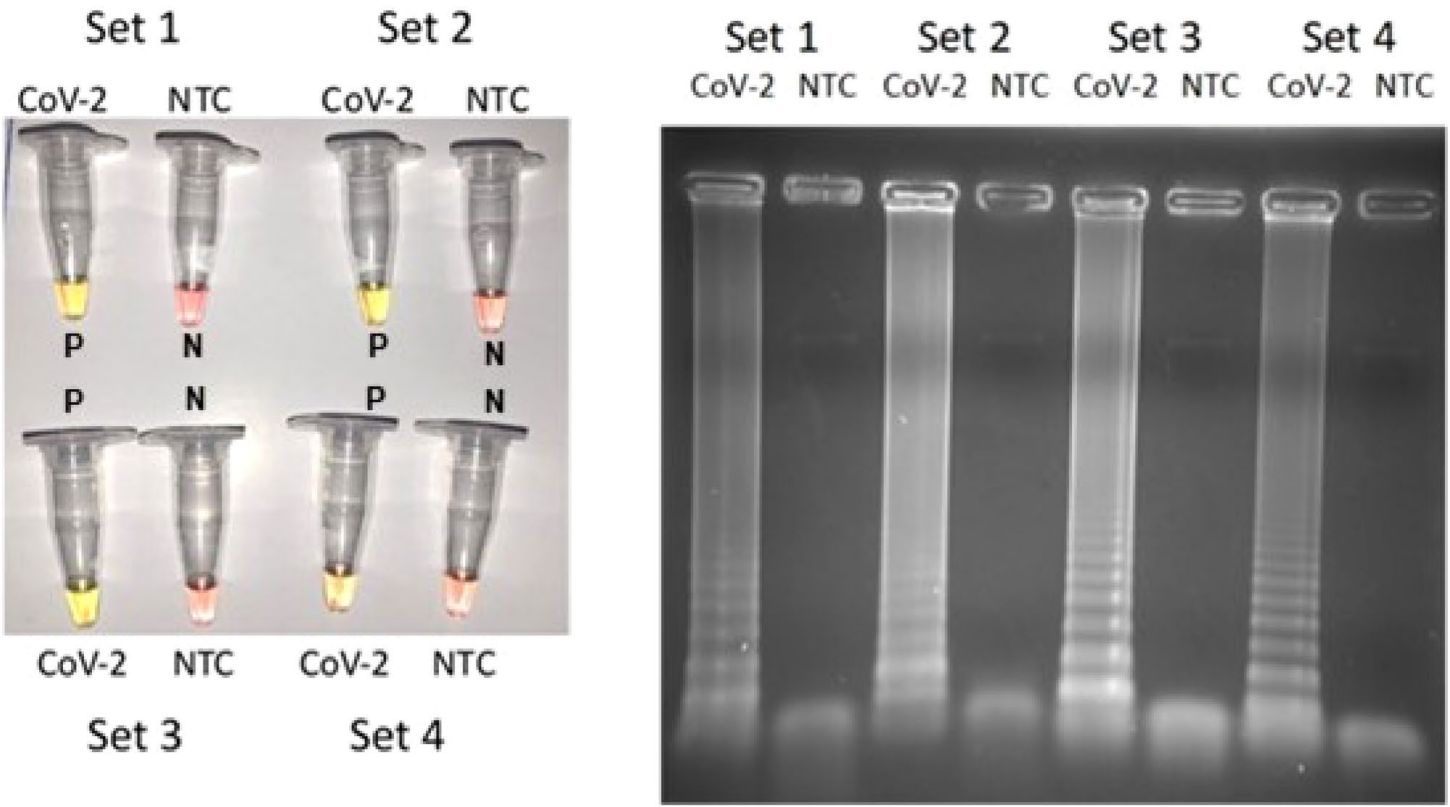
Colorimetric RT-LAMP reaction for SARS-CoV-2 (2400 copies) using four primer sets: (**A)** yellow color (positive reaction indicated as P) and pink color (negative reaction indicated as N). (**B**) Amplification visualized in a 2% agarose gel stained with ethidium bromide, showing characteristic LAMP amplicon profiles in positive samples and no amplification in non-template controls (NTC). Set 1^18^, Set 2^14^, Sets 3 and 4^13^.

We selected three manuscripts that presented promising results^13,14,18^; these sets of primers were evaluated through the viral strain found in Brazil. As described in “Methods”, supernatant RNA extracted from SARS-CoV-2 cultured in Vero cells was quantified by four points in a E-gene standard curve from 50,000 to 50 copies per reaction. We could access a standard curve with amplification efficiency of 98.17% and r^2^ of 0.99, with mean Ct ranging from 24.46 (士0.39) and 34.55 (士0.16). Next, the evaluation of different primers’ set was made to test RT-LAMP analytical sensitivity for SARS-CoV-2 detection. Figure 1 shows the results obtained using four different primers sets in the presence of the quantified virus RNA (2400 copies) or a non-template control (NTC). A tenfold virus RNA template dilution (240 copies) was also performed (data not shown), for primers sets screening accessed by 2 SARS-CoV-2 RNA concentrations. Our chosen criteria to decide which primer set to proceed, were the color intensities in the reaction tubes and in the agarose gel. According to Fig. 1, it is possible to notice that the set three^13^ was slightly more efficient than the other sets due to its color visualization and agarose gel intensity. Set one^18^ and set four^13^ presented a weaker gel visualization when compared with the other sets. After the RT-LAMP reactions it was observed that set three^13^ had a better color contrast between positive and negative samples when compared to Set two^14^.

**Figure 2:**
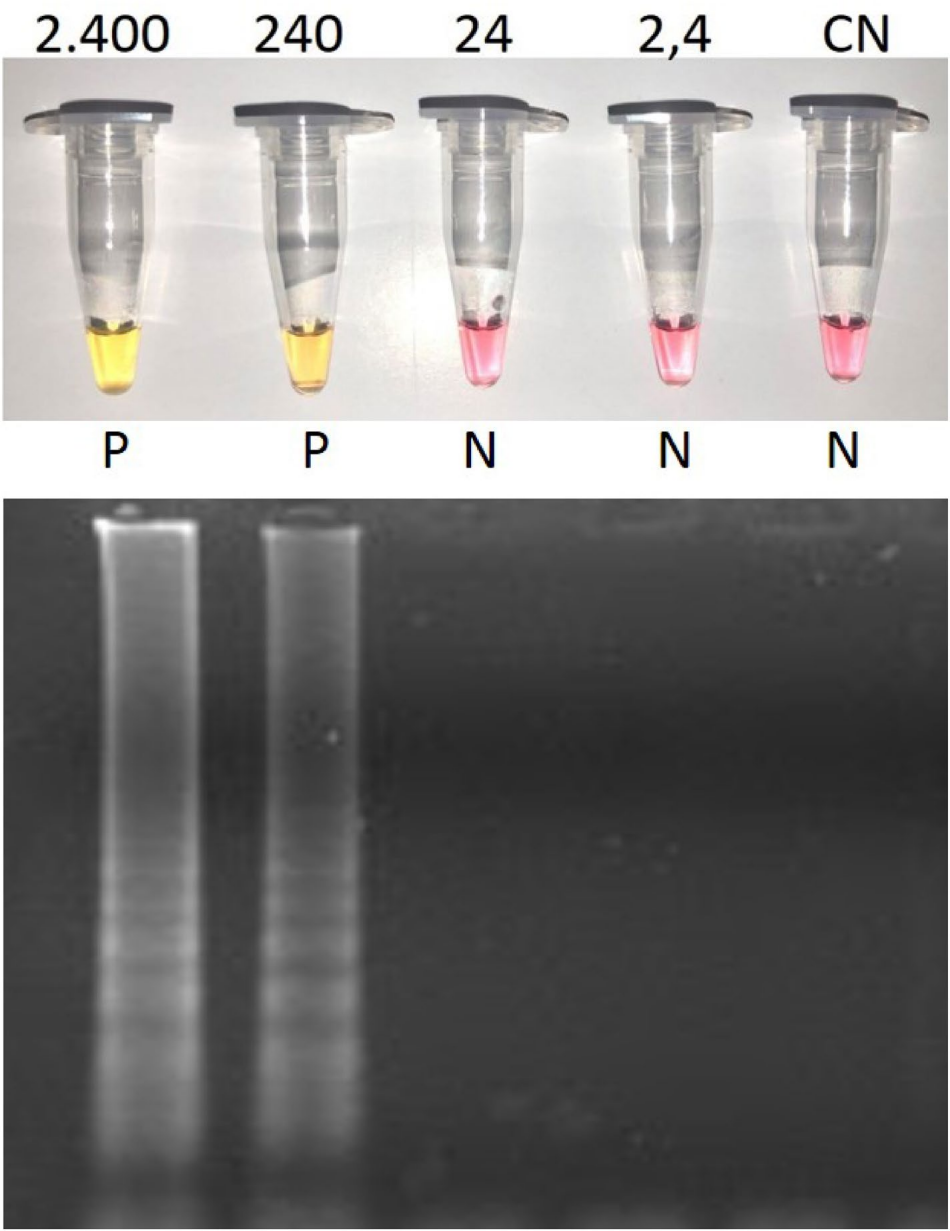
The colorimetric and agarose gel detection of SARS-CoV-2 using RT-LAMP diluting a sample with 2400 copies of SARS-CoV-2 cultured in Vero cells (30 min, 65 °C, and primer set 3). Pink = negative (N); yellow = positive (P); CN = negative control.

Even though it is believed that LAMP primers are easily designed by programs like PrimerExplorer, primer-dimers’ interactions are recurrent^19^. The inner primers (FIP and BIP) are prone to form hairpin structures, and as a consequence the primers fold back on themselves; thus, the high number of primers (usually six) increases the chances of primer-dimer formation^19^. Therefore, it is important to increase the stress on the system in order to evaluate the occurrence of these interactions, preventing the false-positive results. To assess a more precise analytical sensitivity of the reaction (primers set 3^13^), a tenfold SARS-CoV-2 RNA dilution from 2.400 to 2,4 viral genome copies per reaction was made. Figure 2 shows both 2.400 and 240 copies with a yellow color whereas 24 and 2,4 remained pink, as observed in negative control. This performance was also confirmed by visualization in 2% agarose gel stained with ethidium bromide. This experiment shows that the analytical sensitivity was between 240 and 24 copies, which represent an amount clinically significant and relevant for this method.

### Method sensitivity

**Figure 3:**
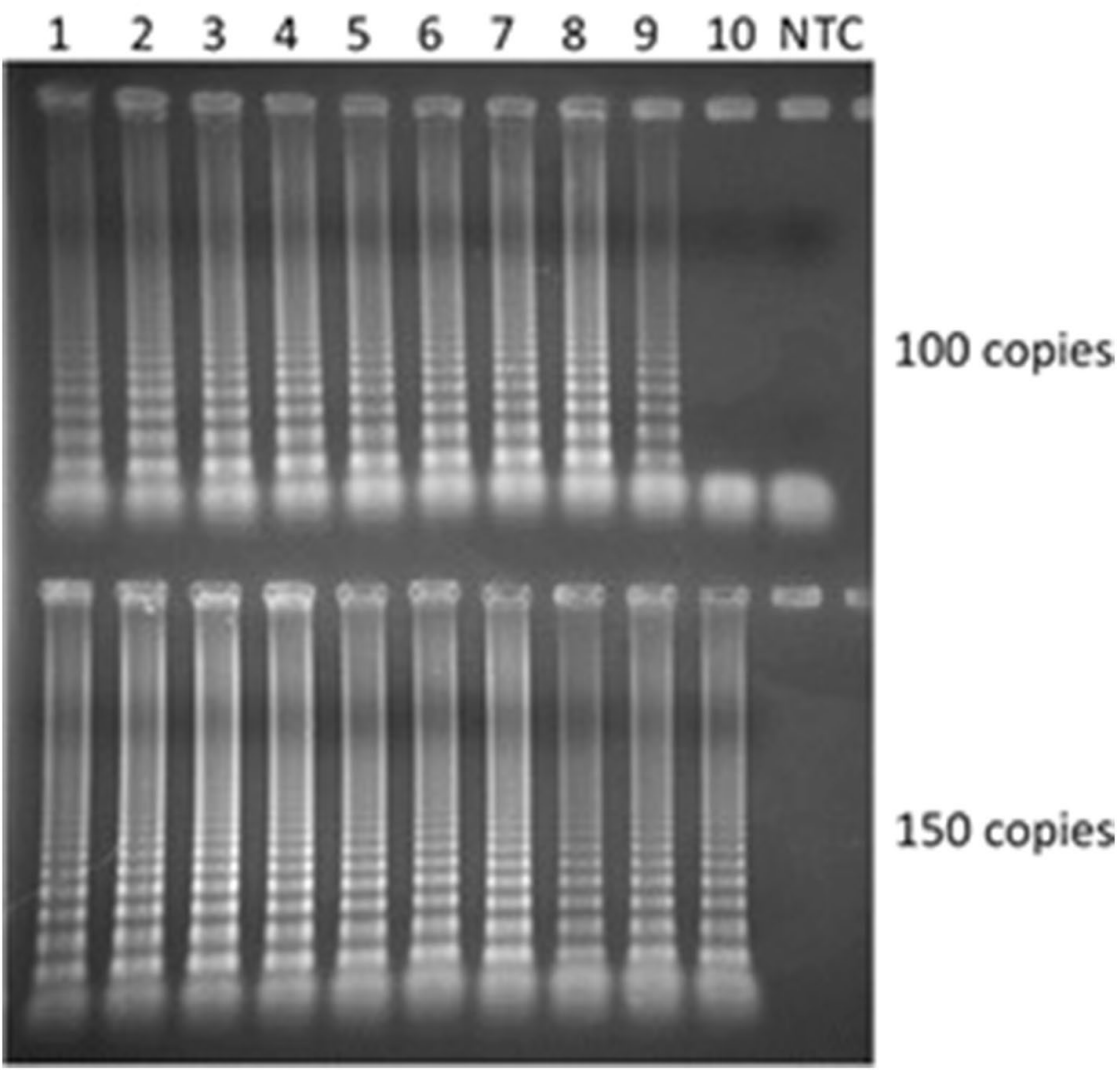
Electrophoresis in 2% agarose gel stained with ethidium bromide and 10 samples of SARS-CoV-2 genome copies and amplified using the colorimetric RT-LAMP reaction at 65 °C for 30 min. All the samples containing 150 copies (100%) and 90% of the samples with 100 copies were amplified.

To achieve a more precise analytical sensitivity level, we further diluted the RNA using 10 different vials containing 200, 150, and 100 copies per reaction. In the samples with 200 copies, all 10 of the vials turned yellow; consequently, 100% were positive. Using 150 and 100 copies per reaction, 90% and 70%, respectively, changed color to yellow. When the amplification products were observed in agarose gel (Fig. 3), 100% amplification was seen in those containing 150 copies, and 90% amplification was seen when 100 copies were presented. With this, we can observe that with 100 copies presented the colorimetric RT-LAMP reaction is positive by agarose gel visualization in mostly reactions but with no color change, probably due to reduced amplicon formation and consequently minimal pH reduction. This demonstrates that RT-LAMP is a sensitive method for SARS-CoV-2 identification; however, for colorimetric diagnosis this sensitivity is lower, representing a methodological weakness.

**Figure 4:**
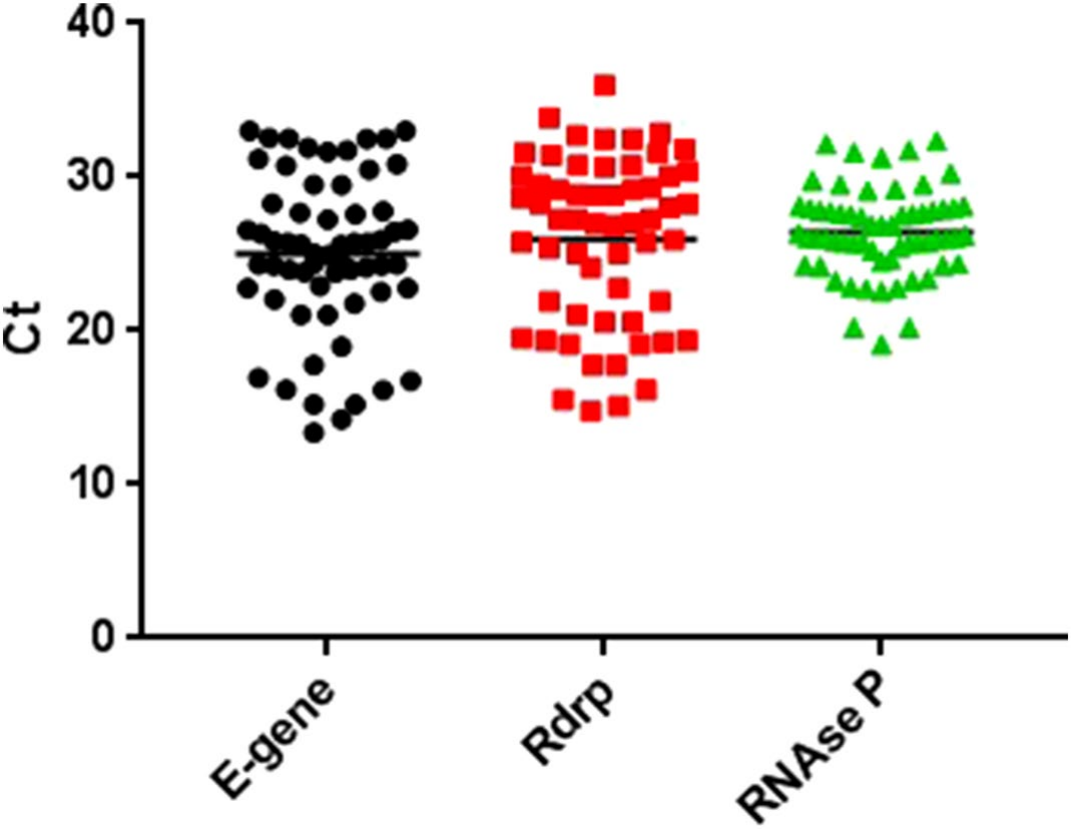
RT-qPCR samples Ct values for E-gene, RdRp gene and RNAse P (internal control).

**Figure 5:**
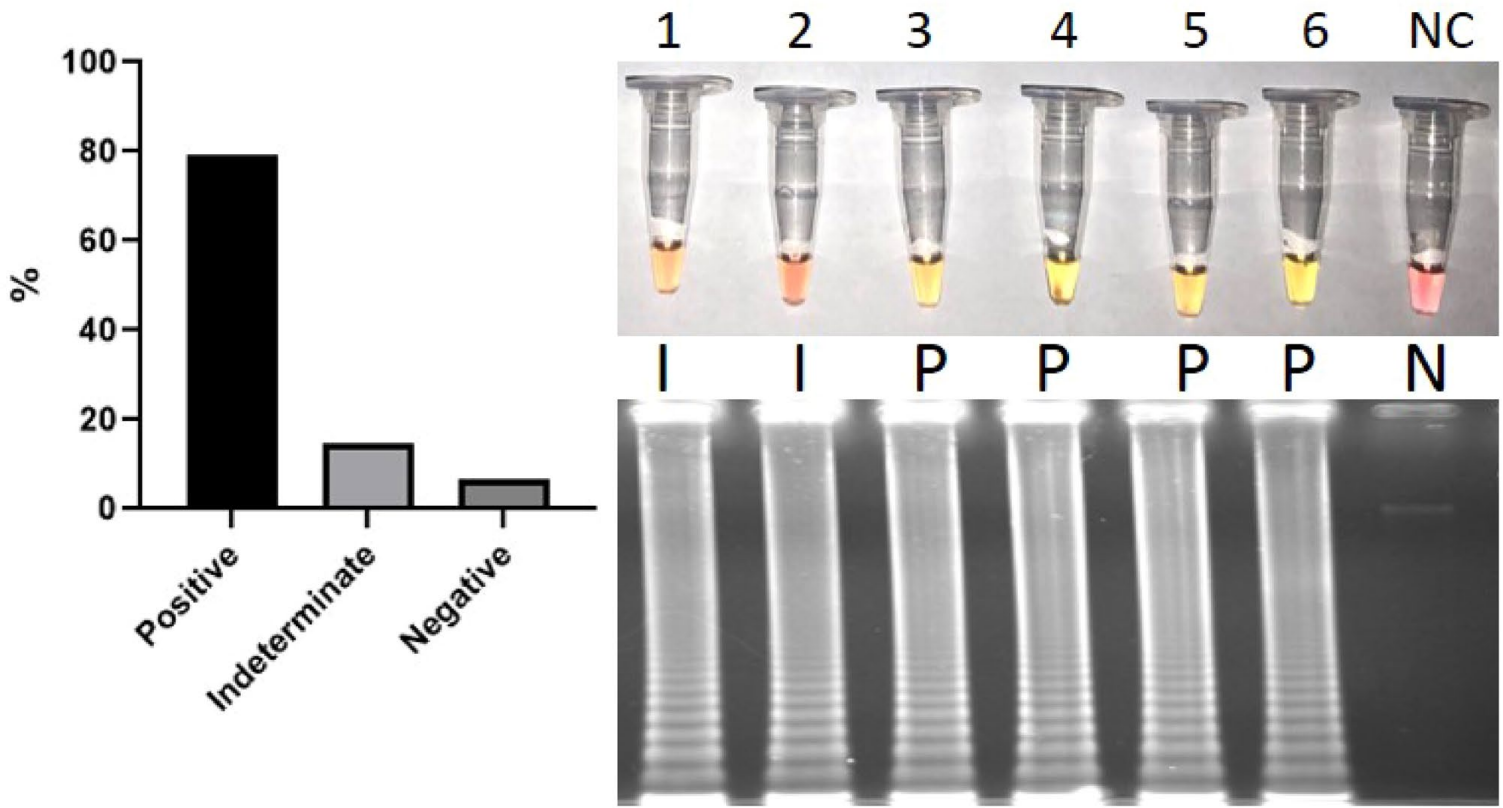
Left panel: Diagnostic sensitivity of colorimetric RT-LAMP with 62 SARS-CoV-2 RT-qPCR positive samples: 79% positive (indicated as P), 14.5% indeterminate (I) and 6.5% negative (N). Right panel: Representative figure of SARS-CoV-2 clinical samples as indeterminate (1 and 2), positive (3 to 6) and a negative control (NC) using the colorimetric RT-LAMP method. The respective amplification product was also visualized in 2% agarose gel stained with ethidium bromide, showing the characteristic LAMP amplicons.

For evaluation of colorimetric RT-LAMP diagnostic sensitivity, we used 62 SARS-CoV-2 clinical samples confirmed by RT-qPCR, with Ct values for the E gene and RdRp gene ranging from 13.38 to 33.00 and from 15.12 to 32.86, respectively. All samples had Ct for internal control RNAse P less than 33 (Fig. 4). When these samples were performed in colorimetric RT-LAMP we observed 79% as positive, 14.5% as indeterminate, and 6.5% negative (Fig. 5). All positive and indeterminate samples, independent of colorimetric RT-LAMP result, had amplification products when visualized in agarose gel, demonstrating that color change may not occur despite amplification. It is important to point out that false-negative results in colorimetric RT-LAMP were obtained in 6.5% of samples but again, with amplification visualized in 2% agarose gel, this result was probably due to lower amplicon formation and consequent low pH reduction. Figure 5 also shows the colorimetric RT-LAMP and 2% agarose gel electrophoresis stained with ethidium bromide of 2 indeterminate and 4 positive SARS-CoV-2 samples. More important, all 49 colorimetric RT-LAMP positive samples had E gene and RdRp RT-qPCR Ct lower than 30, while all nine indeterminate and four negative colorimetric RT-LAMP samples had RT-qPCR Ct for both genes above 30. This is a significant finding because all samples with high viral load, here demonstrated by Ct < 30, were colorimetric SARS-CoV-2 positive, while samples with low viral load, Ct > 30, presented as indeterminate or negative.

### Method specificity

**Figure 6:**
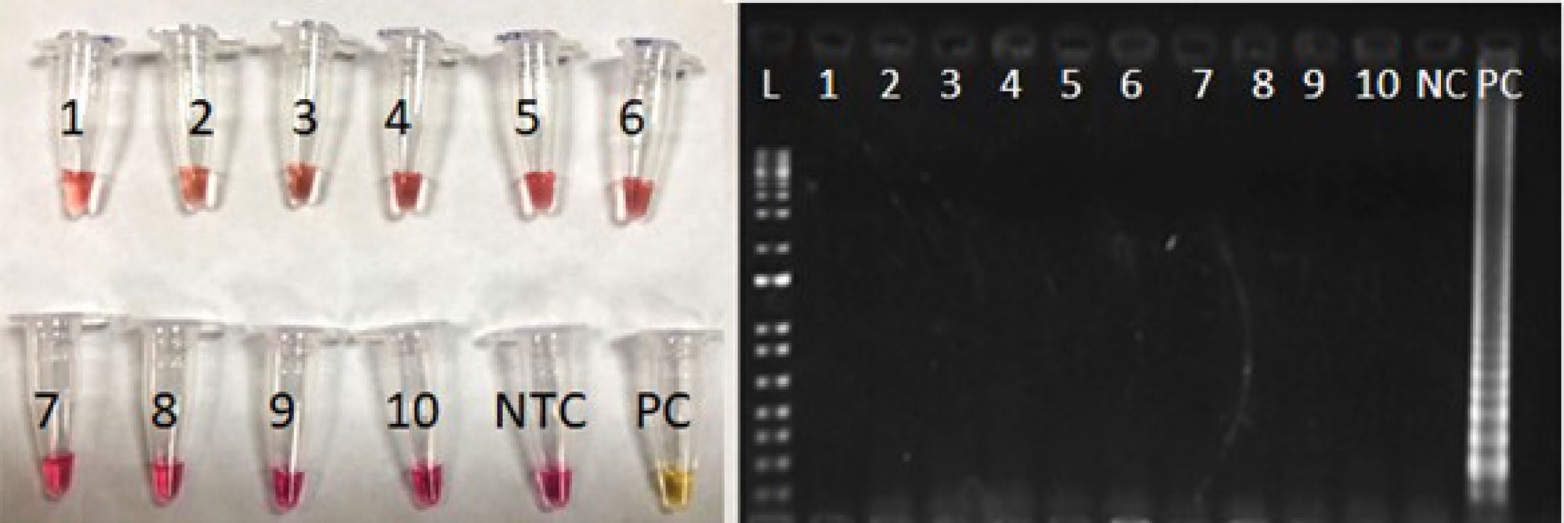
Representative Figure for non-SARS-CoV-2 samples present in Table 1. Samples: 1 and 2: FluA H1N1; 3 and 4: FluB Yamagata; 5 and 6: hCoV-229E; 7 and 8: hCoV-OC43; 9 and 10: hCoV-NL63; NTC: Non-template control; PC: Positive control; L: 1 kb Plus DNA ladder (ThermoFisher). As expected, only the PC changed color to yellow and was amplified in the agarose gel.

Unlike in other reports that used a majority of COVID-19-related samples to evaluate the specificity of the test, we explored a wide range of respiratory viruses that could lead to false-positive results. In this direction, we increased the stress on the test using 131 non-SARS-CoV-2 samples that included 74 healthy subjects and 57 samples with 11 other virus infections, including other coronaviruses. All these samples were collected and diagnosed by the Paraná Central Laboratory (LACEN) in the years 2018 and 2019, i.e., before the SARS-CoV-2 spread in Brazil. Nevertheless, to confirm that these samples were not infected with SARS-CoV-2, all of them were tested using RT-qPCR as described, with no SARS-CoV-2 detection by E-gene or RdRp, and internal control detected in all samples with Ct less than 32.53 (Table 1). When these certified non-SARS-CoV-2 samples were tested with the colorimetric RT-LAMP, no positive reaction was observed either by color change or by agarose gel stained with ethidium bromide, indicating the high specificity of the method (Fig. 6) and representing 100% of diagnostic specificity.

**Table 1:** COVID-19-related samples, showing sample number and diagnostic, RT-qPCR Ct values for RnaseP (internal control) and RdRp and E-gene for SARS-CoV-2 detection.

Number of samples analyzed	Clinical sample	RNase P (internal control) Ct	Genes RdRp e E-gene for SARS-CoV-2
7	Influenza A (FLUA) H1N1	22,85–26,23	Not detected
8	Influenza B (FLUB) Yamagata and Victoria	20,88–31,64	Not detected
5	Virus Parainfluenza 1 (PiV1)	22,43–31,03	Not detected
5	Virus Parainfluenza 2 (PiV2)	20,26–27,00	Not detected
5	Virus Parainfluenza 3 (PiV3)	22,38–28,21	Not detected
6	Adenovirus	22,68–27,67	Not detected
5	Human Coronavirus (hCoV) 229E	24,66–30,60	Not detected
4	Human Coronavirus (hCoV) OC43	23,83–26,68	Not detected
4	Human Coronavirus (hCoV) NL63	24,91–28,51	Not detected
4	Human bocavirus	22,97–28,15	Not detected
4	Respiratory syncytial virus	24,76–29,82	Not detected
74	Health subject	24,74–32,53	Not detected

### Colorimetric quantification

**Figure 7:**
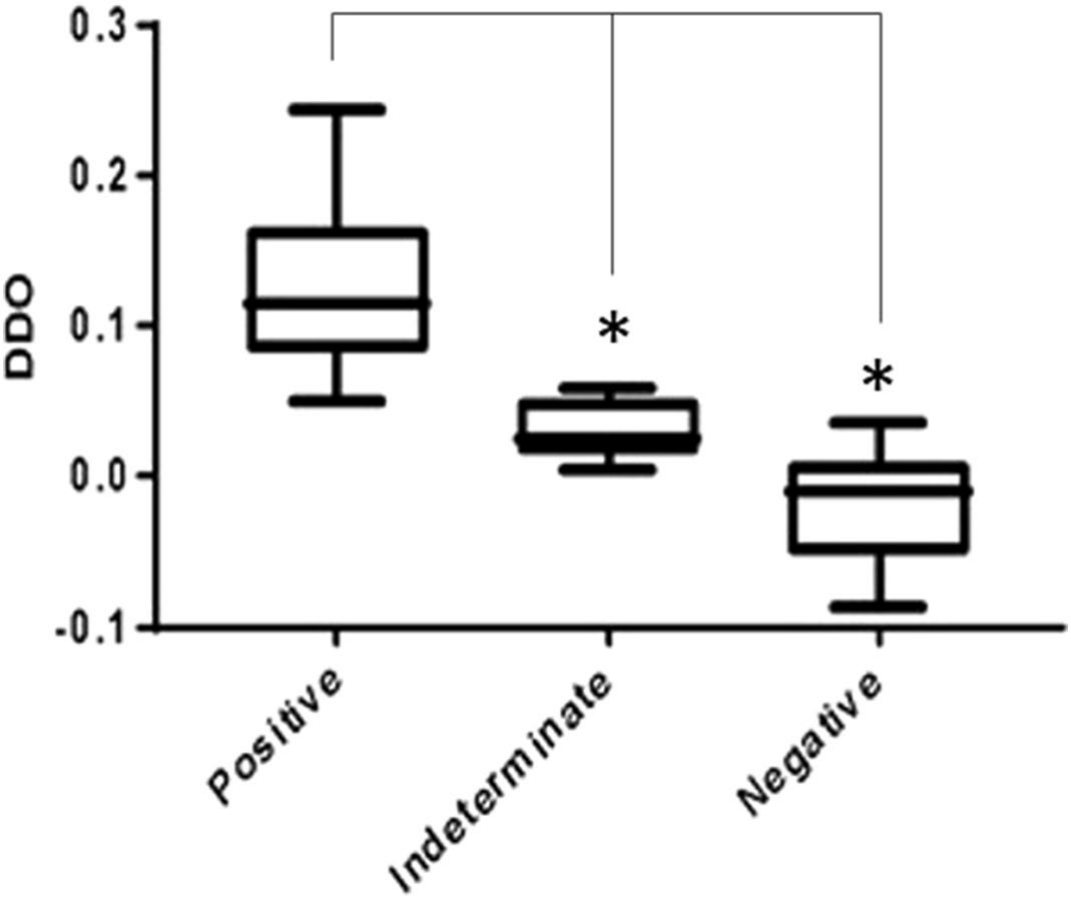
Delta DO for colorimetric RT-LAMP clinical samples measured by spectrophotometry clustering positive, indeterminate and negative results. Statistical significance (*p* < 0,05) was observed in ΔDO between three clusters.

In order to understand the lower diagnostic sensitivity (79%) obtained by us in comparison with that of other authors, we decided to have an objective color determination by calculating the differences between the absorbance values at 434 nm and 560 nm. Through this spectrophotometric analysis we observed three separated clusters: positive, indeterminate and negative (Fig. 7), not just two as described in the overall literature.

All SARS-CoV-2 colorimetric RT-LAMP scientific reports up to the present time consider only positive and negative results when assessing the diagnostic sensitivity; this interpretation may erroneously increase the real sensitivity. If our samples clustered as indeterminate were designated as positive, our diagnostic sensitivity level would reach 93.5% (74% from positive samples + 14.5% from indeterminate samples), a result consistent with other authors. However, it is important to mention that the colorimetric RT-LAMP reaction is designed to be read by the naked eye: indeterminate samples should not be considered in this context.

According to Thi et al.^12^ results, SARS-CoV-2 positive samples with RT-qPCR Ct > 30 maintained the pink color pattern or took more than 30 min to start changing color, determining an affordable limit for colorimetric RT-LAMP. In our study, some SARS-CoV-2 positive samples with RT-qPCR Ct > 30 presented neither yellow nor pink, but an orange color, here clustered as indeterminate. These results could be interpreted as false negatives or false positives by the naked-eye analysis. Therefore, this group of samples is subject to wrong diagnostic treatment or to no treatment at all, thus spreading the virus to others. A colorimetric RT-LAMP as a point-of-care test would not have access to the patient RT-qPCR result as a confirmatory test; therefore, an indeterminate sample could be misinterpreted and biased.

## Discussion

Approaches to detect SARS-CoV-2 based on RT-LAMP have been used broadly as an alternative point-of-care test to detect this disease, including the testing of clinical samples^13,14^, with reaction time varying between 15 and 40 minutes. For example, Yu et al.^14^ obtained a sensitivity of 97.6% with 43 samples, while Zhang et al.^13^ obtained 100% using seven samples. In these two pioneer studies, phenol red was used as the pH indicator to identify positive and negative results by the naked eye, as positive samples turn yellow and the negative ones remain pink. Lamb et al^18^ designed highly specific primers based on the GenBank MN908947 sequence and successfully detected 27 strains of COVID-19 from various locations, which presented no cross reaction with three non-SARS-CoV-2 viruses. Lalli et al.^20^ tested saliva for RT-LAMP with no RNA extraction and reached more than 90% of sensitivity. This is an interesting result since it indicates that the use of saliva associated with RT-LAMP is also possible for the RT-LAMP method. Fowler et al.^21^ demonstrated an overall RT-LAMP sensitivity of 97%, but with a 33 Ct cut-off the sensitivity increased to 100%. They also evaluated the RT-LAMP method without RNA extraction, with dilutions from saliva and directly from the swab, and achieved a sensitivity of 67%.

Despite we demonstrated a lower colorimetric RT-LAMP positive rate (79%) in comparison with other results, for instance those obtained by Wei et al.^22^ (100% with crude samples), L’Helgouach et al.^23^ (95,7% with saliva samples), and Haq et al.^24^ (100% with extracted samples), with Ct values in RT-qPCR less than 30 we achieved 100% specificity with non-SARS-CoV-2 samples, including related virus infections. Few papers explored diagnostic specificity with other viral infections and with as few clinical samples as we did. Nawattanapaiboon et al.^25^, for example, tested colorimetric RT-LAMP for SARS-CoV-2 method with 13 viruses extracted from clinical samples and showed no cross-reaction. Baek et al.^26^ also obtained 100% specificity testing four related coronaviruses, seven human infection influenza viruses, and six other respiratory disease-causing viruses. Just Chow et al.^27^ accessed a large number of other viral infections, looking for 143 other viruses in RT-LAMP for 90 minutes and showed 100% of specificity. Diagnostic specificity is one of the key parameters to validate a diagnostic assay and is necessary because false positive results can lead to different treatments. When LAMP primers are well designed, considering specific and conserved regions, they tend to be specific to the chosen target^28,29^. Many studies design LAMP primers for the N gene, due to its conserved region. Rohaim et al.^30^, however, designed primers for the RdRp gene and obtained no cross-reactivity with other coronaviruses or four other respiratory viruses. Our results show the high specificity of the colorimetric RT-LAMP test for COVID-19 even when it is exposed to 11 species of other respiratory related viruses. This high diagnostic specificity of the method shows that colorimetric RT-LAMP can be an efficient and reliable tool to SARS-CoV-2 diagnostic during the early stages of the disease, which is essential to control the pandemic.

Although RT-qPCR is the gold-standard method to detect COVID-19, it has become evident that this technology is too laborious to effectively stop the virus from spreading. On the other hand, the use of lateral flow tests used to scan the populations has presented high rates of false positives and negatives, becoming a cause of concern instead of a solution. In this context, new technologies need to be introduced to meet the testing demand. The colorimetric RT-LAMP method, described by a few authors as being able to detect SARS-CoV-2, has been evidenced to combine simplicity and accuracy, thereby presenting a viable alternative to fight this disease. Our results show that targeting the N gene with the colorimetric RT-LAMP is a sensitive and specific approach to detect SARS-CoV-2 if the reaction runs at 65°C for 30 minutes. We also proved that the method is robust, stressing the test with 11 respiratory-related viruses. We also report here that the color changes depend on the viral load on the sample. These indeterminate samples represent a delicate cluster that should be carefully analyzed to return a confident result. Nonscientific paper report SARS-CoV-2 colorimetric RT-LAMP results in three clusters described here as positive, indeterminate, and negative. We demonstrated that the color change is not evident and easily distinguished by the naked-eye in an indeterminate cluster, correlating with viral load accessed by RT-qPCR. Corroborating this data, Thi et al.^12^ demonstrated that SARS-CoV-2 detection by colorimetric RT-LAMP is directly dependent on viral load and showed that positive samples with a RT-qPCR Ct<30 changed color within the first 30 minutes of reaction. Samples with RT-qPCR Ct > 30 either did not change color or did so at time points >35 min, simultaneously with a color change observed in some of the negative samples. But increasing the time is not helpful since nonspecific amplification reactions may occur. A robust color change in the colorimetric RT-LAMP test for samples with RT-qPCR Ct values between 30 and 35 was observed for only one of 10 samples. This finding suggests a limit for colorimetric RT-LAMP assay corresponding to a Ct ≈ 30. Nawattanapaiboon et al.^25^ using RT-qPCR, determined 47 SARS-CoV-2 positive samples with Ct values ranging from 17 to 38, with sensitivity of 95.74% and specificity of 99.95%. A colorimetric RT-LAMP false-negative sample exhibited high RT-qPCR Ct values of 34.17 and 34.93 for ORF1ab- and N-specific primers, respectively. Furthermore, all RT-qPCR SARS-CoV-2 positive samples that yielded Ct values lower than 33 were found by the authors to be true positive in colorimetric RT-LAMP. However, when the Ct values were greater than 33, the percentages of true positive samples decreased to 60% and 40%, for RT-qPCR ORF1ab and N primers, respectively. Again, Schermer et al.^31^ using colorimetric RT-LAMP failed to detect the virus in specimens that were positive in diagnostic RT-qPCR at Ct values > 30 for E and S gene. It is important to note that all these experiments apparently have been conducted with RT-qPCR validated clinical samples, while colorimetric RT-LAMP as a point-of-care test will be performed alone. Therefore, clinical samples with a lower viral load, here demonstrated by RT-qPCR Ct > 30, may produce false-negative or indeterminate results. More importantly, all these authors ignored the indeterminate sample color that we demonstrated to exist, especially with correlation between RT-qPCR results. For application of colorimetric RT-LAMP for point-of-care purposes, we strongly recommend its use for samples with Ct<30 or in acute cases, as it has been observed in this study the technique detection limit.

In short, our results corroborate the data from other authors, showing that colorimetric RT-LAMP can be used as a first-line method for fast and reliable COVID-19 detection. However, color change interpretation should be carefully analyzed since it represents an essential property for the assay performance as a point-of-care tool. Pink color should be considered negative, yellow as positive, and orange as indeterminate. We also verified that the patient viral load is an important limitation on colorimetric RT-LAMP for point-of-care usage, as we demonstrated that RT-qPCR > 30 may return indeterminate or false-negative results. We here suggest that when the color change is not clearly distinguishable, the sample should be repeated or directed to RT-qPCR, and the patient should stay in quarantine meanwhile. We also observed that RT-LAMP will be more effective if used in the first week after the symptoms appear^13,14,16^. In short, colorimetric RT-LAMP is a sensitive and specific method that can be used to fight the new coronavirus pandemic if the method limitations are understood and respected.

## Methods

### Samples and ethical statement

Supernatant from SARS-CoV-2 cultured in Vero (ATCC, United States) cells was extracted with QIAamp RNA Viral Mini Kit (Qiagen, Germany), following the manufacturer’s instructions. The RNA and consequent viral genome quantification was performed by real-time PCR on LightCycler 96 (Roche, Germany) using an E-gene standard curve (SARS-CoV Frankfurt1; Full virus RNA, Lot2; Institute of Virology, Charité) in three independent experiments, based on Corman et al.^17^ All the clinical samples were obtained from the Paraná Central Laboratory, Curitiba, Brazil, after obtaining both the respective and Fiocruz ethics committee’s evaluation and approval. All samples collection and experimental conduction were carried out in accordance with guidelines and Brazilian regulations. All recruited patients have read, discussed, and signed an informed consent before sample collection. For clinical sensitivity we used 62 positive SARS-CoV-2 samples, and for specificity we used 57 respiratory related-disease virus samples and 74 healthy samples, totaling 193 samples. All samples had the RNA extracted with MagNa Pure 96 (Roche, Germany) kit and analyzed in the LightCycler 96 (Roche, Germany) thermocycler.

### Real-time quantitative PCR

RT-qPCR was the gold-standard diagnostic tool used to test the clinical samples with the Corman et al. protocol^17^, using 5 μL of RNA, 12.5 μL of 2X reaction buffer and 1 μL of reverse transcriptase. For E gene amplification were used 400 nM of the primer E_Sarbeco_F, 200 nM of E_Sarbeco_P1 and 400 nM of E_Sarbeco_R. The concentration of RdRp gene primers were 600 nM for RdRp_SARSr-F, 100 nM for RdRp_SARSr-P2 and 800 nM for RdRp_SARSr-R, in a final reaction volume of 20 μL. All primers and probes for the SARS-CoV-2 E-gene, RdRp and human RNase P (internal control) were purchased from Integrated DNA Technologies (IDT, United States). All the reactions were carried out using SuperScript III Platinum One-Step qRT-PCR Kit (ThermoFisher, United States) at 50 °C for 30 min, 95 °C for 5 min, 45 cycles of 95 °C for 15 s, and 58 °C for 30 s using the LightCycler 96 (Roche, Germany). A SARS-CoV-2 positive sample detected by RT-qPCR was expected to present a threshold cycle (Ct) less than 35 in both the targets. All the samples also had to amplify the internal control RNase P at a maximum Ct of 35 to procedure validation.

### RT-LAMP primers

Previously published studies have demonstrated the use of a wide range of SARS-CoV-2 RT-LAMP primer sets providing high-quality performance. Of those, we chose to reproduce the primers designed by Zhang et al., Yu et al. and Lamb et al.^13,14,18^ due to the high quality of the data presented. We selected and named four (one to four) primer sets to compare their performance levels (Supplementary Table S1). Zhang et al.^13^ designed a primer set for the N and ORF region of SARS-CoV-2, and Yu et al.^14^ targets the ORF1ab region. Lamb et al.^18^, however, does not specify the target region of their primer set. All the primer were purchased from Integrated DNA Technologies (IDT, United States) and resuspended in at a concentration of 100 μM in nuclease-free water (Invitrogen, United States).

### RT-LAMP assays

For the colorimetric RT-LAMP reaction, the WarmStart Colorimetric LAMP 2X Master Mix (NEB, England) was used. This kit contains phenol red, a pH indicator that changes color from pink to yellow due to the formation of pyrophosphate ions produced during amplification. Each set of primers contained FIP (Forward Inner Primer) and BIP (Backward Inner Primer) at 1,6 μM each, FOP (Forward Outer Primer) and BOP (Backward Outer Primer) at 0,2 μM each, and FL and BL (Forward Loop and Backward Loop) at 0,4 μM each. The reaction was conducted with a final volume of 25μL, using 12,5μL of WarmStart Colorimetric LAMP 2X Master Mix, 6μL of Primer Oligo, 3μL of RNA, and 3,5μL of RNase Free water. Primers from set one were incubated at 63 °C, while those of sets two, three, and four were incubated at 65 °C, all for 30 min. After this step, all the sets were incubated at 80 °C for 10 min for enzyme inactivation. All the incubations were done using the ProFlex PCR System (Applied Biosystems, United States). Positive reactions presented a yellow color while the negative ones remained pink. Orange samples were considered indeterminate. Human RNA (oncological breast cancer cell line MCF7) was used as negative control (NC). For amplification confirmation, reactions were run on 2% agarose gel electrophoresis (100 V) for 45 min, stained with ethidium bromide and visualized using a UV transilluminator (L-Pix Chemi, Loccus, Brazil).

### Colorimetric quantification of the RT-LAMP reaction

To quantify the colorimetric RT-LAMP results, 20 µL of each sample after the reactions were measured using a 384-well black plate at 434 nm and 560 nm (Synergy H1—BioTek), which are the maximum absorbance values for the two forms of the dye, i.e., yellow and pink, at pH 6 and 8, respectively. In case of positive reactions, the pink color decreases in intensity and the yellow absorbance increases. Therefore, a positive delta is observed by subtracting the yellow absorbance from the pink one (ΔDO i.e., 434 nm—560 nm). Statistical analyze was performed with ΔDO for positive, indeterminate and negative samples by GraphPad Prisma using parametric t-test, with *p* < 0,05.

### Data availability

The datasets and raw data generated during and/or analyzed during the current study are available from the corresponding author on reasonable request.

## Supplementary Information

Supplementary Tables.
