# Supplementary material for: Colorimetric RT-LAMP SARS-CoV-2 diagnostic sensitivity relies on color interpretation and viral load
Source: Sci Rep. 2021 Apr 27;11:9026. doi: 10.1038/s41598-021-88506-y (PMC8079700; doi:10.1038/s41598-021-88506-y)
Supplement: Supplementary file 1 — Supplementary Tables. [file 41598_2021_88506_MOESM1_ESM.docx]

# Colorimetric RT-LAMP SARS-CoV-2 Diagnostic Sensitivity Relies on Color Interpretation and Viral Load

# Mateus Nóbrega Aoki1,+, Bruna de Oliveira Coelho1,+, Luiz Gustavo Bentim Góes2,3, Paola Minoprio2, Edison Luiz Durigon2,3, Luis Gustavo Morello1, Fabricio Klerynton Marchini1, Irina Natassja Riediger5, Maria do Carmo Debur5, Helder Nakaya2,4 and Lucas Blanes1*

1 Laboratory for Applied Science and Technology in Health, Carlos Chagas Institute, Oswaldo Cruz Foundation (Fiocruz), Curitiba, 81310-020, Brazil.

2 Scientific Platform Pasteur - University of São Paulo, São Paulo, 05508-020, Brazil.

3 Departamento de Microbiologia – ICB‐II, Universidade de São Paulo, São Paulo, 05508-000, Brazil.

4 Department of Clinical and Toxicological Analyses, School of Pharmaceutical Sciences, University of São Paulo, São Paulo, 05508-000, Brazil.

5 Paraná´s Central Laboratory (LACEN-PR), Curitiba, 80045-150, Brazil.

*lucas.blanes@fiocruz.br

+these authors contributed equally to this work

| **Primers sequence** | | **Target region** | **Reference** |
| --- | --- | --- | --- |
| **Set 1** | FIP: AGAGCAGCAGAAGTGGCACAGGTGATTGTGAAGAAGAAGAG  BIP: TCAACCTGAAGAAGAGCAAGAACTGATTGTCCTCACTGCC  FOP: TCCAGATGAGGATGAAGAAGA  BOP: AGTCTGAACAACTGGTGTAAG  FL: CTCATATTGAGTTGATGGCTCA  BL: ACAAACTGTTGGTCAACAAGAC | Not informed | Lamb et al |
| **Set 2** | FIP: AGGTGAGGGTTTTCTACATCACTATATTGGAACAAGCAAATTCTATGG  BIP: AGGTGAGGGTTTTCTACATCACTATATTGGAACAAGCAAATTCTATGG  FOP: CCACTAGAGGAGCTACTGTA  BOP: TGACAAGCTACAACACGT  FL: CAGTTTTTAACATGTTGTGCCAACC  BL: TAGAGCCATGCCTAACATGCT | ORF1ab | Yu et al |
| **Set 3** | FIP: TCTGGCCCAGTTCCTAGGTAGTCCAGACGAATTCGTGGTGG  BIP: AGACGGCATCATATGGGTTGCACGGGTGCCAATGTGATCT  FOP: TGGCTACTACCGAAGAGCT  BOP: TGCAGCATTGTTAGCAGGAT  FL: GGACTGAGATCTTTCATTTTACCGT  BL: ACTGAGGGAGCCTTGAATACA | Gene N | Zhang et al |
| **Set 4** | FIP: GAGGGACAAGGACACCAAGTGTATGGTTGAGCTGGTAGCAA  BIP: CCAGTGGCTTACCGCAAGGTTTTAGATCGGCGCCGTAAC  FOP: CTGCACCTCATGGTCATGTT  BOP: AGCTCGTCGCCTAAGTCAA  FL: CCGTACTGAATGCCTTCGAGT  BL: TTCGTAAGAACGGTAATAAAGGAGC | ORF | Zhang et al |

Supplementary Table S1: Four primers sets initially used, with sequence, SARS-CoV-2 target region (when informed) and reference.
